# Hope and Resilience During a Pandemic Among Three Cultural Groups in Israel: The Second Wave of Covid-19

**DOI:** 10.3389/fpsyg.2021.637349

**Published:** 2021-02-18

**Authors:** Orna Braun-Lewensohn, Sarah Abu-Kaf, Tehila Kalagy

**Affiliations:** ^1^Conflict Management & Resolution Program, Department of Interdisciplinary Studies, Ben-Gurion University of the Negev, Beer Sheva, Israel; ^2^Department of Public Policy & Management, Guilford Glazer Faculty of Business and Management, Ben-Gurion University of the Negev, Beer Sheva, Israel

**Keywords:** hope, sense of coherence, resilience, stress, pandemic, ethnic groups

## Abstract

The aim of this study was to explore the coping resources of hope and sense of coherence, which are rooted in positive-psychology theory, as potential resilience factors that might reduce the emotional distress experienced by adults from three cultural groups in Israel during the chronic-stress situation of a pandemic. The three cultural groups examined were secular Jews, Ultra-Orthodox Jews, and Arabs. We compared these cultural groups during the second wave of the Covid-19 pandemic, just before the Jewish New Year (mid-September 2020) as a second lockdown was announced. Data were gathered from 248 secular Jews, 243 Ultra-Orthodox Jews, and 203 Arabs, who were 18–70 years old (*M* = 37.14, *SD* = 12.62). The participants filled out self-reported questionnaires including the Brief Symptom Inventory as a measure of emotional/psychological distress (i.e., somatization, depression, and anxiety) and questionnaires about sense of coherence and different types of hope (i.e., intrapersonal, interpersonal, and transpersonal) as measures of coping resources and resiliency. Differences were found between the three groups in terms of several variables. The Arab participants reported the highest levels of emotional distress and the lowest levels of interpersonal and transpersonal hope; whereas the Ultra-Orthodox participants revealed the highest levels of sense of coherence and other resilience factors. A structural equation model revealed that, in addition to the sociodemographic factors, only sense of coherence and intrapersonal hope played significant roles in explaining emotional distress, explaining 60% of the reported distress among secular Jews, 41% among Ultra-Orthodox Jews, and 48% among Arabs. We discuss our findings in light of the salutogenic and hope theories. We will also discuss their relevancy to meaning-seeking and self-transcendence theory in the three cultural groups.

## Introduction

The Covid-19 pandemic has led governments of many countries around the world to impose lockdowns that have lasted several weeks to several months. In Israel, the first lockdown lasted from March to May 2020. Once Israel got out of the first lockdown, the feeling among citizens was that normal life had returned. Requests from the government to minimize gatherings were not enforced and schools were opened, with students in isolated groups (capsules), at the beginning of September. Thus, by mid-September, a second wave had arrived and, just before the Jewish New Year, the government announced a second lockdown, with restrictions that were more severe than those imposed during the first wave of the epidemic.

Against the backdrop of the second wave and the second lockdown, the present study aimed to assess two main resources, hope and sense of coherence (SOC), as potential resiliency factors capable of reducing three common emotional problems (i.e., somatization, anxiety, and depression) during this difficult period. Based on the hope theory of Snyder (2000) and salutogenic theory (Antonovsky, 1987), which are inherently related to the concepts of meaning-seeking and self-transcendence, we wanted to examine the above-mentioned resiliency factors and emotional-distress variables among three main cultural groups in Israel: secular Jews, Ultra-Orthodox Jews, and Arabs. We sought to explore similarities and differences among these groups, in terms of the different variables as well as the ways in which resiliency factors explain emotional distress under the conditions of chronic stress imposed by an ongoing epidemic.

### Hope

Hope for the future enables effective coping with developmental challenges. It elaborates options for individuals and helps them to examine sources of personal strength by relating to the future and not only relying on the past (Sharabi et al., 2012). Several researchers have tried to define hope and have emphasized different components of this construct. Snyder (2002) offered a definition in which hope combines individual perceptions, in order to generate alternative ways to achieve desirable goals. Others have suggested that hope is a personal resource that can be developed and fostered and which is essential for coping, success, and decision-making.

Researchers have agreed that hope involves emotional elements of expectation, as well as cognitive and deductive thinking to pursue new ideas and solutions (Lazarus, 1991; Snyder, 1994). Hope is seen by some researchers as a positive attitude toward life and the ability to have optimistic views (Moorey and Greer, 1989; Strang and Strang, 2001; Sawatzky et al., 2009). Hope is based on high-level cognitive processing, which requires mental representations of positively valued, abstract future situations. More specifically, it requires setting goals, planning how to achieve those goals, and the use of imagery and creativity, cognitive flexibility, mental exploration of novel situations, and even risk-taking (Lazarus, 1991; Snyder, 1994, 2000). The affective component of hope is considered to be a consequence of cognitive elements and may contain positive, as well as negative features since individuals may realize that the achievement of their goal may involve struggles, costs, and endurance (Snyder, 1994, 2000). It seems that the emotional element of hope is rooted in early experiences of trust, which are influenced by others and by external events (Erikson et al., 1994).

While Snyder (1994, 2000) emphasized the cognitive component of hope, Jacoby and Goldzweig (2014) elaborated on Snyder's concept of hope and identified three sub-concepts that emphasize the emotional components of hope, in addition to the cognitive component. In their view, the term *intrapersonal hope* refers to hope in which a person looks into him/herself while assessing his/her resources. *Interpersonal hope* refers to the relationships one has with different significant and meaningful individuals in whom one can trust. *Transpersonal hope* refers to hope that relies on supernatural powers and which gives an individual a sense of meaning and purpose (Jacoby and Goldzweig, 2014). The representation of hope in this way is connected to the concept of self-transcendence, which also represents consciousness that is related to various sources from one's internal self to one's environment and broadening to include the cosmos (Wong, 2016).

In the current study, we evaluated the three components of hope as resiliency factors that might be involved in reducing psychological/emotional distress. Indeed, one recent study has shown that hope, defined as expectation from the future, leads to positive outcomes even during a pandemic, meaning that a sense of leading a meaningful life mediates the role of basic health in predicting stress resulting from Covid-19 (Trzebiński et al., 2020; Walsh, 2020; Yildirim and Arslan, 2020).

### Salutogenesis and Sense of Coherence

Antonovsky (1979) proposed the conceptualization and model of *salutogenesis*, which means the “origin of health,” in the stress and health field. Rather than classifying health/illness dichotomously, this continuum model suggests that each individual is somewhere on the ease/dis-ease continuum at any given moment (Antonovsky, 1987). According to this model, people have general resistance resources that can help them to conceptualize the world as organized and understandable. SOC represents the motivation and the internal and external resources one can use to cope with stressors and plays an important role in the way one perceives challenges throughout life. SOC is a global orientation to see the world as more or less comprehensible (the internal and the external world are perceived as rational, understandable, consistent, and expected), manageable (the individual believes that s/he has the necessary resources available to deal with situations), and meaningful (the motivation to cope and the commitment to emotionally invest in the coping process; Antonovsky, 1987). Both the cognitive aspects of SOC and the emotional-motivational aspects reflect major components of the concept of self-transcendence. In the self-transcendence process, it is claimed that cognitive restructuring processes lead to choices and outcomes (Wong, 2016), which can also be viewed as indicative of comprehensibility and manageability. Additionally, an inherent tendency to move beyond one's own self-interest and become aware of sources of purpose (Wong, 2016) can be viewed in light of the salutogenic concept of meaningfulness.

The salutogenic model suggests that an individual with a strong SOC is less likely than one with a weak SOC to perceive many stressful situations as threatening and, therefore, anxiety-provoking. Given their tendency to perceive the world as meaningful and manageable, individuals with a strong SOC will be less likely to feel threatened by stressful events, including a pandemic, and, therefore, will be less vulnerable to developing psychological distress in such a situation (Gómez-Salgado et al., 2020; Ruiz-Frutos et al., 2020; Schäfer et al., 2020).

Research in the stress literature dealing with the concept of SOC and its application to mental-health problems has differentiated between acute stress and chronic stress. It has been argued that the resource of SOC is more influential and plays a powerful role in reducing stress in the context of chronic stress, as opposed to acute stress (Sagy and Braun-Lewensohn, 2009). In the present study, we suggest that the situation we are examining is a chronic-stress situation, as the pandemic has been with us since February–March of 2019.

### Psychological Distress in a Chronic-Stress Situation

Stress encompasses cognitive, emotional, physical/somatic, and social variables (Lazarus and Folkman, 1984). Research conducted during normal times among a representative sample of Israeli adults to identify baseline levels of various psychological problems such as anxiety, depression, and somatization found very low levels of each of those problems (Gilbar and Ben-Zur, 2002). However, research conducted during various stressful situations has shown that individuals who are exposed to stressful situations, including a pandemic, tend to be especially vulnerable to developing common psychological problems, including somatization, anxiety, and depression. More specifically, the lockdowns and curfews that were implemented during the Covid-19 pandemic influenced psychological problems and distress, including anxiety and depression, as noted in several recent studies (Hossain et al., 2020; Lei et al., 2020).

### Demographic Characteristics

#### Gender

In various contexts and countries, the resiliency factors of SOC and hope have been analyzed with regard to gender and no significant gender differences have been found for either variable (Roothman et al., 2003; Maree et al., 2008; Van Schalkwyk and Rothmann, 2008; Braun-Lewensohn and Mosseri Rubin, 2014). In general (Nolen-Hoeksema, 2001; Gronning et al., 2018), and during the COVID-19 pandemic specifically, women have been shown to be more likely to develop psychological problems and distress, including anxiety, depression, and somatization (Liu et al., 2020; Sfendla and Hadrya, 2020).

#### Age

Antonovsky (1987) claimed that SOC continues to develop until the age of 30, at which point it stabilizes. However, several studies from the last decade have claimed that SOC continues to develop and strengthen during adulthood (beyond age 30) and declines as individuals approach old age (Nilsson et al., 2010; Braun-Lewensohn and Sagy, 2014). Similarly, the hope construct is strengthened during adulthood and weakens in old age (Bailey and Snyder, 2007).

As for the outcome variables of psychological distress, several studies have suggested that during stressful events younger adults are more exposed to social media and news, which results in more anxiety, depression, and somatization. This has also been found in the context of the current pandemic (Qiu et al., 2020; Sfendla and Hadrya, 2020).

#### Socio-Economic Status (SES)

SES is among the general resiliency resources that Antonovsky (1987) suggested are important for the development of a strong SOC. Indeed, several studies have demonstrated that the higher individuals' socio-economic status, including education and income, the stronger their SOC (Volanen et al., 2004). Similarly, low SES was found to be associated with lower levels of hope; whereas high SES was found to be associated with higher levels of hope (Snyder, 2002). Finally, low-SES individuals have been found to report more psychological problems such as anxiety, depression, and somatization, as compared to their high-SES counterparts (De Mello et al., 2013) also during the COVID-19 pandemic (Patel et al., 2020).

### Cultural Groups Examined in This Study

#### Israeli Jews

Diversity in Israel includes not only the variety of ethnicities that constitute the country's overall population, but also the cultural variety within the Jewish majority, a significant proportion of which (more than 30%) is made up of immigrants. A third of the Jewish population defines itself religiously as “traditional”; whereas another third defines itself as “religious,” or “very religious” (Bistrov and Sofer, 2010). Overall, Jewish Israeli society is considered a Western, individualistic society (Sagy et al., 2001).

#### Ultra-Orthodox Society

The population of Ultra-Orthodox Jews in Israel numbers about 1,125,000, accounting for ~12.5% of the country's population (Cahaner and Malach, 2019). They represent a significant minority group in Israeli society. The Ultra-Orthodox do not differ from the majority group in terms of race or nationality, but are separated by ideological, religious, and social motivations, which unite them (Brown, 2017). Researchers of Ultra-Orthodox society usually divide this sector into three main groups: Hasidic, Lithuanian (*Misnagdim*), and Mizrahi (Brown, 2017). However, in a general sense, when relating to these three groups, we may point to two characteristics common to all of them: Torah study as a constitutive value and social isolation. These two values are central to these communities. However, in recent years, social and economic changes have stretched the boundaries of Ultra-Orthodox communities and the effects of these changes are still unfolding (Braun-Lewensohn and Kalagy, 2019).

The Ultra-Orthodox community can be characterized as a religious collectivist community, with very high levels of social capital relative to other populations in Israeli society. Consequently, the Ultra-Orthodox individual is surrounded by three concentric groups: the family, the community, and the public. These three circles provide members of the Ultra-Orthodox community with physical and spiritual support, alongside demands for the normative behavior accepted in the community (Bart and Ben-Ari, 2015). The social capital of this community has been written about in the research literature in the contexts of health and mental well-being. Tchernichovsky and Sharoni (2015) found that the health of the Ultra-Orthodox population is better than would be expected based on their socio-economic status. Among this population, social capital affects health mainly through psychosocial support, including community aid, which reduces mental stress. This is despite the fact that it does not seem that this population has any greater access to medical services or organizations, as compared to other groups in Israel (Tchernichovsky and Sharoni, 2015).

#### Arabs in Israel

The Arab minority comprises about 21% of the entire Israeli population and includes Muslims (83%), Christians (9%), and Druze (8%; Gharrah, 2012; Central Bureau of Statistics (Israel), 2020). Arab society is highly collectivistic-patriarchal, although it is currently undergoing a rapid process of transition (Al-Haj, 1988; Haj Yahia-Abu Ahmad, 2006; Azaiza, 2013). In this context, inequalities based on gender and age (and, in recent years, also education) are very common (Al-Haj, 1988; Hofstede and Hofstede, 2005). This minority society differs from Jewish Israeli society in that it is less individualistic and more authoritarian and in its emphasis on connectedness and social relationships with meaningful others in one's social environment (Haj Yahia, 2019). Arabs also differ from the Jewish majority in terms of language, religion, and other cultural factors (Al-Krenawi et al., 2009). Arabs in Israel are a largely disadvantaged minority with major determinants of mental-health problems, including political, social, cultural, and economic factors (Abu-Kaf, 2019). The Arab minority is subject to various forms of discrimination that may contribute to social and economic disparities between them and the Jewish majority (Ghanem, 2001; Okun and Friedlander, 2005; Pinson, 2007; Yiftachel, 2009; Knesset Research and Information Center, 2016). The Arab community suffers from poverty, harsh living conditions, violence, discrimination and stigma, and poor employment and working conditions (Keinan and Bar, 2007; The Galilee Society-The Arab National Society for Health Research and Services, 2013; Central Bureau of Statistics, 2015; Knesset Research and Information Center, 2015).

The above-mentioned cultural groups have been examined in various studies with regard to the variables examined the present study. Due to numerous SES factors, in addition to their national status, Arabs in Israel have been found to suffer from more psychological problems and distress (e.g., depression, somatization, and anxiety) than their Jewish counterparts (Haberfeld and Cohen, 2007; Baron-Epel et al., 2010; Abu-Kaf, 2019). As for the other variables, it has been found that Ultra-Orthodox individuals express the strongest SOC, followed by secular Jews, and then Israeli Arabs (e.g., Abu-Kaf et al., 2017; Kalagy et al., 2017). The picture is more complicated when comparing secular Jews and Bedouin Arabs in terms of various factors of hope. In a previous study, Bedouin Arabs reported stronger collective hope than Secular Jews, but no differences were found between those two groups in terms of individual hope. As for the explanation of numerous psychological problems by SOC and hope, while SOC was found to be the strongest predictor of a lack of psychological symptoms among Jews, hope was found to be the strongest among Arabs (Braun-Lewensohn and Sagy, 2010).

In this study, we sought to explore the prevalence of the major resiliency factors of SOC and hope, as well as psychological distress (in terms of anxiety, depression, and somatization) among three cultural groups in Israel (i.e., secular Jews, Ultra-Orthodox Jews, and Arabs). We compared these variables among these groups and estimated the contributions of the resiliency factors to the explanation of the reported psychological distress. We further evaluated the roles of the sociodemographic variables of gender, age, and SES in explaining psychological distress during the long-term Covid-19 pandemic. Based on the salutogenic model (Antonovsky, 1987) and the hope theory (Snyder, 2000), we hypothesized that individuals with strong SOC and high levels of intrapersonal, interpersonal, and transpersonal hope would be more resilient and, therefore, would react with less anxiety, depression, and somatization (Gómez-Salgado et al., 2020; Trzebiński et al., 2020). We further hypothesized that women, low-SES individuals, and older individuals would be more vulnerable during the pandemic and that those factors would contribute to higher levels of stress (De Mello et al., 2013; Sfendla and Hadrya, 2020).

## Materials and Methods

### Participants

Data were gathered from 248 secular Jews (Age: *M* = 39.76, *SD* = 13.58), 243 Ultra-Orthodox Jews (Age: *M* = 37.50, *SD* = 12.70), and 203 Arabs (Age: *M* = 33.95, *SD* = 10.46), who were all 18–70 years old (*F* = 12.50, *p* < 0.001). Women accounted for 50.4% of the secular group (*n* = 113), 53.4% of the Ultra-Orthodox group (*n* = 118), and 57.9% of the Arab group (*n* = 106; **χ**^2^ = 2.22, *p* = 0.32). SES was evaluated in terms of three levels: below-average salary, average salary, and above-average salary. In our sample, a below-average salary was reported by 118 (52.7%) of the secular participants, 147 (66.5%) of the Ultra-Orthodox participants, and 126 (68.9%) of the Arab participants. An average salary was reported by 55 (24.6%) of the secular participants, 37 (16.7%) of the Ultra-Orthodox participants, and 32 (17.5%) of the Arab participants. An above-average salary was reported by 51 (22.8%) of the secular participants, 37 (16.7%) of the Ultra-Orthodox participants, and 25 (13.7%) of the Arab participants (**χ**^2^ = 14.27, *p* < 0.01).

### Procedure

All of the ethical guidelines applicable to this study were followed. The study was approved by the Human Subjects Ethics Committee of the Conflict Management and Resolution Program at Ben-Gurion University of the Negev (Approved Ethics Form No. 2020-008). Participants completed self-report questionnaires in mid-September 2020, just before the Jewish New Year, as a second curfew was being announced and after ~6 months of the Covid-19 pandemic. The participants were recruited by the midgam panel (https://www.midgampanel.com/) and were informed that the researchers were interested in their experiences. They were also informed that their participation was voluntary and anonymous and that they were free to withdraw their participation for any reason at any time during the questionnaire procedure.

### Measures

#### Demographics

Participants were asked to report their gender, age, and SES. SES was measured by one question in which participants were presented the average salary in Israel and had to report whether they earn below the Israeli average salary, average salary or above the Israeli average salary.

#### Hope

We used the 18-item, short version of a hope questionnaire (Jacoby and Goldzweig, 2014). Each item was scored on a Likert scale ranging from 1 (*do not agree at all*) to 4 (*totally agree*). In addition to a global scale of hope, this questionnaire included three subscales: interpersonal hope (five items; i.e., *I draw strength from the relationships in my life*), intrapersonal hope (nine items; i.e., *At difficult times in my life, I trust myself that I will be able to get out of the difficult situation*), and transpersonal hope (four items; i.e., *I have a belief that gives me a sense of comfort*). In the present study, the mean of the relevant items was computed for each subscale. The Cronbach's alpha coefficient was α = 0.88 for both the intrapersonal scale and the transpersonal scale. For the interpersonal scale, it was α = 0.83.

#### Sense of Coherence (SOC)

SOC was measured using an instrument (Antonovsky, 1987) that included a series of items scored on a 7-point Likert-type scale that had anchoring phrases at each end. High scores indicate a strong SOC. An account of the development of the SOC scale and its psychometric properties, showing it to be reliable and reasonably valid, appears in Antonovsky (1987, 1993) writings. In this study, SOC was measured using the short-form scale consisting of 13 items, which has been found to be highly correlated to the original long version (Antonovsky, 1993). The scale includes items such as: “*Doing the things you do every day is*” with answers ranging from 1 (*a source of pain and boredom*) to 7 (*a source of deep pleasure and satisfaction*). In the present study, the mean was calculated and Cronbach's alpha coefficient for the scale was 0.82.

#### Symptoms

We used the short version of the Brief Symptom Inventory (Derogatis and Fitzpatrick, 2004), which is comprised of 18 items that are each rated on a 5-point Likert scale (0 – *not at all*; 4 – *very much*). This questionnaire examines three areas of psychological and psychiatric problems: somatization, depression, and anxiety. The reliability of the short version of the questionnaire and its three subscales has been reported to be good (Franke et al., 2017). Here are examples of items from each subscale. Somatization: “*To what extent have you felt faint or experienced dizziness?”* Anxiety: “*To what extent have you suffered from a feeling of stress?”* Depression: “*To what extent have you suffered from a feeling of depression?”* The Cronbach's alpha coefficients for each of the stress indices (i.e., anxiety, depression, and somatization) and for the global severity index were all α = 0.88.

### Statistical Analyses

To address our first objective, frequencies and standard deviations of each variable were computed. The second objective related to the comparison of the three cultural groups and One-way ANOVA was conducted. Finally, to evaluate the model in which the sociodemographic factors of gender, age, and SES and the resiliency factors of SOC and the three dimensions of hope explain the psychological/emotional distress (i.e., anxiety somatization, and depression) in each group, structural equation modeling was conducted using the AMOS 26 program (Arbuckle and Wothke, 1999).

## Results

For most of the stress indices, our results were higher than the community adult Israeli norms (Gilbar and Ben-Zur, 2002). The average scores for all of the resiliency factors, namely, SOC and the various hope scales, were also at the upper end of the scales (Table 1).

**Table 1:** Means and *SD*s of the psychological-distress variables measured in this study, as compared to Israeli norms.

	**Current sample**		**Israeli norm**	
	***N*** **=** **592**		***N*** **=** **510**	
	***M***	***SD***	***M***	***SD***
Somatization	0.63	0.81	0.62	0.68
Depression	0.96	0.90	0.70	0.69
Anxiety	0.98	0.90	0.85	0.71
GSI^a^	0.86	0.78	0.72	0.59

a *GSI, Global Severity Index*.

Significant differences were found among all of the examined variables (see Table 2). The most prevalent differences were found between the Ultra-Orthodox group and the other two groups. In all cases, the Ultra-Orthodox group reported higher levels of resiliency factors and lower levels of psychological distress. Differences were also found between secular Jews and Arabs in two of the three hope scales. Secular Jews reported higher levels of interpersonal hope; whereas Arabs reported higher levels of transpersonal hope. As for psychological distress, a significant difference was found only for somatization, with the Arab group reporting more somatization than the secular group.

**Table 2:** Differences between the groups in terms of the main variables.

	**Secular Jews (a)**		**Ultra-Orthodox Jews (b)**		**Arabs (c)**		***F***
	***N*** **=** **215**		***N*** **=** **214**		***N*** **=** **177**		
	***M***	***SD***	***M***	***SD***	***M***	***SD***	
SOC (1–7)	4.45	0.93	4.82	0.90	4.27	0.88	18.23^***^^(ab, bc)^
Intrapersonal hope (1–4)	3.09	0.54	3.35	0.49	3.17	0.59	12.14^***^^(ab, bc)^
Interpersonal hope (1–4)	3.28	0.62	3.45	0.49	3.08	0.69	16.31^***^^(ab, bc, ac)^
Transpersonal hope (1–4)	2.33	0.77	3.72	0.50	3.13	0.68	231.08^***^^(ab, bc, ac)^
Somatization (0–4)	0.62	0.81	0.44	0.60	0.84	0.91	15.04^***^^(ac, bc)^
Depression (0–4)	1.08	0.94	0.66	0.72	1.15	0.90	18.12^***^^(ab, bc)^
Anxiety (0–4)	1.04	0.97	0.73	0.73	1.16	0.88	12.67^***^^(ab, bc)^

*** *p **<** 0.001*.

**Figure 1:**
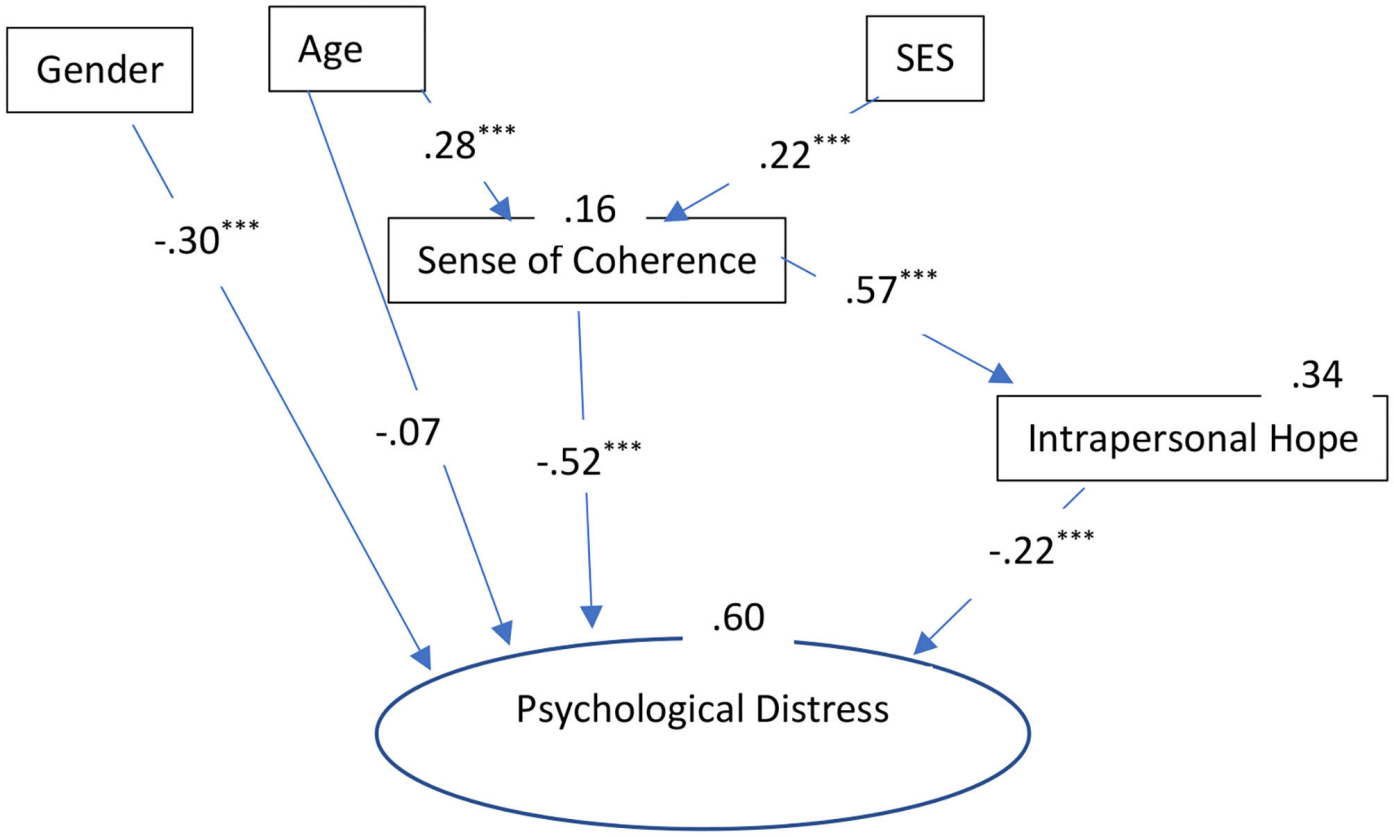
The roles of sociodemographic and resiliency factors in explaining psychological distress: Results of the path analysis for secular Jews. ^***^p **<** 0.001. SES, Socio-Economic Status.

**Figure 2:**
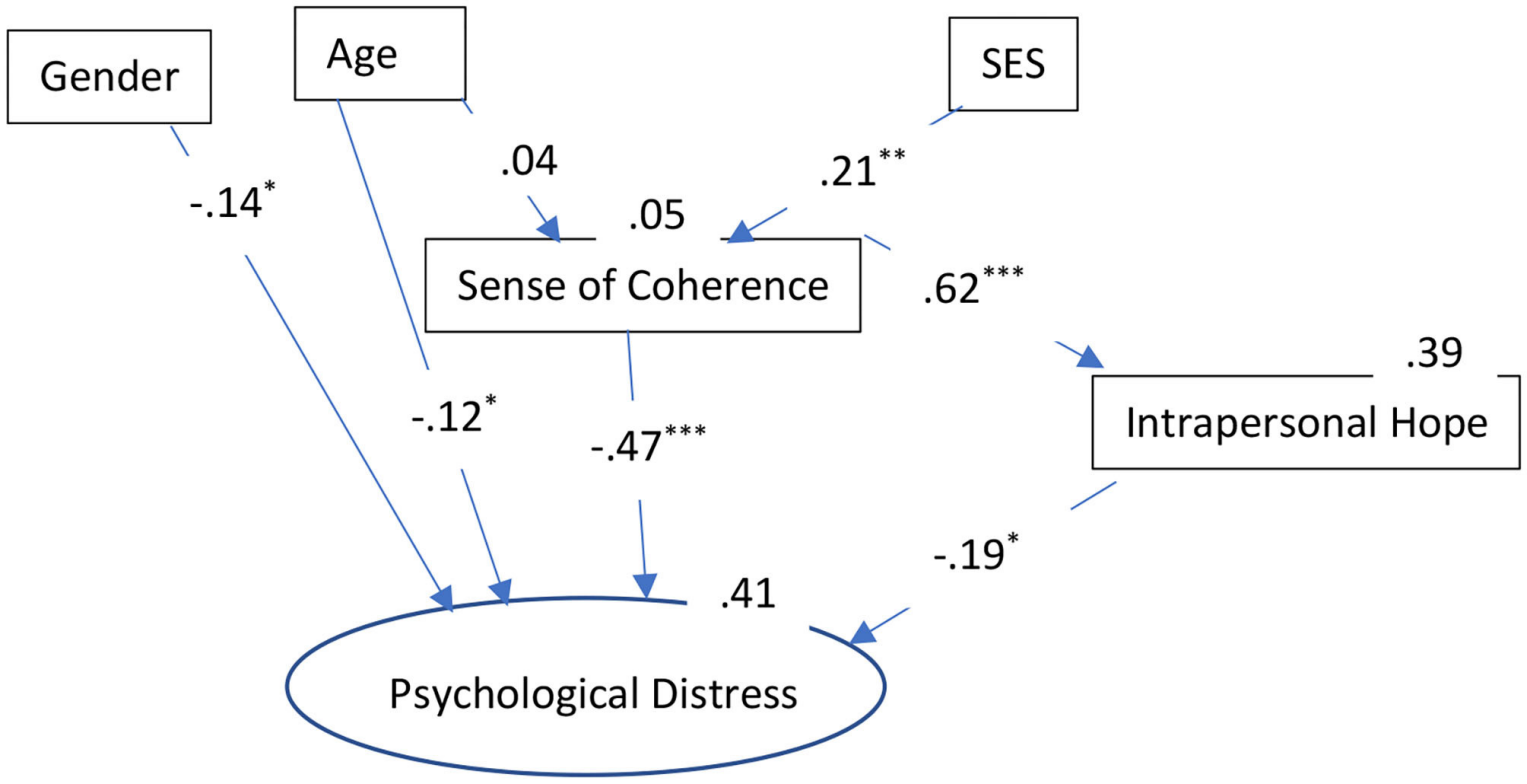
The roles of sociodemographic and resiliency factors in explaining psychological distress: Results of the path analysis for Ultra-Orthodox Jews. ^*^p < 0.05; ^**^p < 0.01; ^***^p < 0.001. SES, Socio-Economic Status.

**Figure 3:**
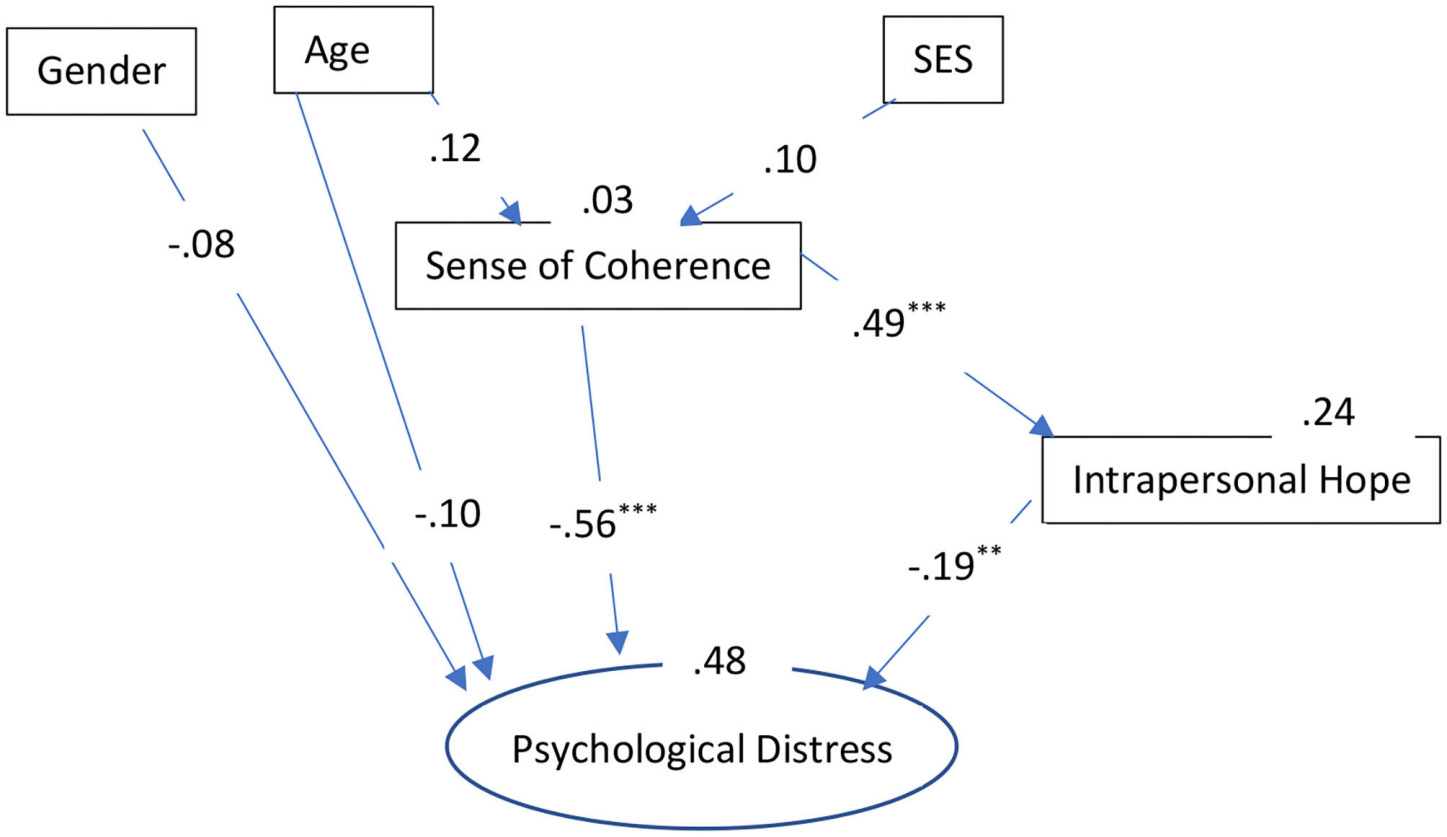
The role of sociodemographic and resiliency factors in explaining psychological distress: Results of the path analysis for Arabs. ^**^p < 0.01; ^***^p < 0.001. SES, Socio-Economic Status.

We used multi-group analysis to compare the effects of the different resiliency factors among each group (i.e., secular Jews, Ultra-Orthodox Jews, and Arabs). The mean for each scale was computed separately and used as a manifest variable. For psychological distress (the dependent variable), a latent variable was created using the three dimensions of stress reactions as indicators (i.e., somatization, depression, and anxiety). Model fit was assessed using the ratio of chi-square to degrees of freedom (**χ**^2^/*df*) incremental fit index (IFI; Bollen, 1989), the comparative fit index (CFI; Bentler, 1990), and the root mean square error of approximation (RMSEA; Browne and Cudeck, 1993). Acceptable fit is indicated by a **χ**^2^/df ratio of 3 or less (Carmines and McIver, 1981), IFI and CFI equal to or >0.90, and RMSEA of <0.08 (Browne and Cudeck, 1993; Hoyle, 1995). The indices were adequate for the overall model: $\chi_{\left(54\right)}^{2}$ = 137.29, *p* < 0.001; **χ**^2^/*df* = 2.86; CFI = 0.95; IFI = 0.95; and RMSEA = 0.05 (Figures 1–3).

The final SEM, presents only significant relationships, as variables that were not significant for the explanation of the stress indices were deleted from the final model. The overall model explained 60% of the variance in psychological distress among the secular Jews, 41% of the variance among the Ultra-Orthodox Jews, and 48% of the variance among the Arabs. The indirect effects of the various variables on psychological distress were as follows. *Secular Jews*: SES (−0.14), age (−0.18), SOC (−0.13); Ultra-Orthodox: SES (−0.12), age (−0.02), SOC (−0.12); and Arabs: SES (−0.07), age (−0.08), SOC (−0.09).

It is important to note that, as for the socio-demographic factors, it is clear that being a women in the secular group had a powerful and significant effect on the development of the examined forms of psychological distress, while it had less of an effect in the Ultra-Orthodox group and no effect in the Arab group. Age also had different effects on psychological distress. In terms of the total direct and indirect effect, age seemed to have the most powerful effect in the secular group (−0.25), followed by the Arab group (−0.18), and, to a lesser extent, in the Ultra-Orthodox group (−0.14); with younger individuals experiencing greater psychological distress. Lastly, SES was also most powerful among the secular group with a total effect (direct and indirect) of −0.14, less powerful among the Ultra-Orthodox group (−0.12), and least powerful among the Arab group (−0.07).

These results indicate that, in all three groups, the resiliency factors were the most prominent in their contributions to the explanation of the psychological distress, with SOC having the strongest effect [secular Jews: total effect (−0.65); Arabs: total effect (−0.64); and Ultra-Orthodox Jews: total effect (−0.59)]. In order to test the differences in the strength of the relationships between the resiliency factors and the dependent variable, the effects of SOC and intrapersonal hope on psychological distress were examined using a nested model. Equality constraints among groups were assigned for each effect, to allow for the comparison of the constrained model with the free model. Statistical differences were found for the variables as follows: SOC and psychological distress [($\chi_{\left(51\right)}^{2}$ = 1056.7); $\Delta \chi_{\left(3\right)}^{2}$ = 919.41; *p* < 0.001] and intrapersonal hope and psychological distress [($\chi_{\left(51\right)}^{2}$ = 595.7); $\Delta \chi_{\left(3\right)}^{2}$ = 458.41; *p* < 0.001]. This means that despite the fact that these variables made important and significant contributions to stress in all three groups, SOC and intrapersonal hope differed significantly in their contributions to psychological distress in the three groups. Both variables had the greatest contribution to psychological distress in the secular group.

## Discussion

The aim of this study was to compare three major cultural groups in Israel against the backdrop of a second lockdown during the Covid-19 pandemic. First, we explored the prevalence of psychological distress (i.e., anxiety, depression, and somatization), as well as resiliency factors among individuals in Israeli society. Second, we examined differences between the cultural groups in terms of the main study variables. Then, through the prism of several resiliency theories (i.e., salutogenesis, hope, and self-transcendence, which are all rooted in positive psychology), we attempted to examine how hope and SOC explain psychological distress in those cultural groups.

Our results show that, overall, during the pandemic, Israelis reported increased psychological distress, as compared to non-crisis times. These results replicate results from countries around the world which also reported elevated stress symptoms (e.g., Husky et al., 2020; Rossi et al., 2020). It seems that the ongoing stressful situation, together with the fact that people were just days before the second lockdown, brought individuals meaningful stress that led to anxiety and depression. Moreover, the fact that people had hardly gotten back to normal life led them to feel that they were giving up major parts of their life. In addition, the fact that this was the second time in which major Jewish holidays were to be celebrated only with the people with whom one lived, as opposed to celebrations with extended families, caused a great deal of distress.

Our second aim was to compare Ultra-Orthodox Jews, secular Jews, and Arabs, in terms of resiliency factors and psychological distress. Overall, it can be stated that the Ultra-Orthodox group reported the strongest resiliency factors and, correspondingly, suffered the fewest symptoms of psychological distress. This could be due to the fact that Ultra-Orthodox individuals have strong faith in God, which leads them to experience a meaningful life, which leads, in turn, to better mental health (Wong, 2019, 2020a). These results might also be a result of the traditional structure of the Ultra-Orthodox family (Cahaner and Malach, 2019). Ultra-Orthodox society is characterized by large families with many children living in dense communities. Feelings of loneliness are less frequent in such environments, which, in turn, leads to lower levels of the types of psychological distress evaluated in this study. Additionally, throughout the years it was found that religious practices and beliefs are positively related to life satisfaction, happiness and other indicators of mental well-being (Koenig, 2001; Guillford, 2002). Thus, it seems that religious belief provides a positive worldview, which gives meaning to experiences, whether positive or negative. This is significant because it provides a sense of purpose in life, an optimistic attitude and a high level of hope (Pargament et al., 2000; Koenig, 2001). In addition, religious beliefs can evoke positive emotions, such as joy, neutralizing or relieving stress in daily life (Koenig, 2001).

From the examination of symptoms of psychological distress, it appears that both the secular Jews and the Arabs experienced high levels of distress. During regular times, members of Arab society in Israel suffer from more psychological distress than Jews, due to their political and economic status (i.e., lower participation in the workforce, lower income, and lower educational attainment), as well as social factors such as inferior social position, social exclusion, and an intense conflictual relationship with the Jewish majority (Kaplan et al., 2010; Abu-Kaf, 2019; Abu-Kaf et al., 2020). It should be noted that also around the world, during the COVID-19 pandemic, minorities including Muslim Arabs reported elevated levels of stress (Miconi et al., 2020). However, the significance of our results is that during this pandemic, it seems that secular Jewish society closed this gap with regards to psychological distress. Strong distrust in elected officials and representatives and their decisions, which appeared to the public to be random and based not on the actual situation on the ground, but rather on political pressure, led individuals to feelings of helplessness, which turned into distress.

Our results can be interpreted through the lens of the second wave of positive psychology (PP.2). This wave explores the negative sides of life while highlighting its optimistic parts and focusing on the productive functioning of individuals (Mayer et al., 2019; Mayer and Vanderheiden, 2020). Therefore, in spite of the fact, that negative outcomes of psychological distress have emerged as result of the health pandemic, it could be, that it is only a first stage in which individuals can explore their situation and grow out of these difficulties which will transform also to positive outcomes.

Our main objective related to the explanation of psychological distress (i.e., somatization, anxiety, and depression) by the various demographic and resiliency factors (i.e., SOC and hope) and the examination of differences between the three cultural groups. Overall, in all three groups, the main resiliency factors of SOC and hope explained psychological distress significantly and powerfully. It should be mentioned that among the various components of hope, only the intrapersonal component was significant in all three groups.

Likewise other studies during the COVID-19 pandemic (e.g., Barni et al., 2020) the most significant and meaningful factor that most powerfully explained the psychological/emotional distress in the three cultural groups was SOC, with its three dimensions of comprehensibility, manageability, and meaningfulness.

Similarly, the self-transcendence theory, which is the meaningfulness dimension of SOC, has seemed to be the most important component across studies (Eriksson and Mittelmark, 2017). Thus, it seems that creating meaning for life, aside from being able to comprehend and manage one's life even during the chronic-stress situation of a pandemic, helps individuals from various cultures and backgrounds to better handle the situation, and, therefore, to suffer from less psychological distress. These results add significant knowledge to that provided by previous research, which compared acute and chronic situations of political violence and found that in chronic-stress situations among Western secular cultures, SOC serves as a major protective factor against psychological distress (Sagy and Braun-Lewensohn, 2009).

The second important factor to explain psychological distress in the current study was intrapersonal hope, which means turning into oneself in order to assess one's resources. Our results elaborate understanding relating to basic and global hope during pandemic (Trzebiński et al., 2020). Intrapersonal hope is connected to self-transcendence and allows the individual to be conscious of his/her internal resources and to apply those resources in the wider environment. Thus, it seems that positive, hopeful thoughts serve as significant protective factor in the face of the chronic stress of a pandemic.

In light of PP 2.0 it seems that those individuals who felt suffering as the starting point, were able to discover ways in which they adopt to the situation and transform their pain, distress and suffer to positive elements such as hope and SOC, which in turn result in wellbeing and strengths (Wong, 2020b). This processes of turning pain and suffer into growth and strength might be similar to the relationships which can be found throughout research between post-traumatic stress and post-traumatic growth (i.e., Liu et al., 2017).

A striking result emerged from our examination of the effect of gender on psychological distress. Similarly to results from populations around the world (Spoorthy et al., 2020), being a secular woman meant being more vulnerable to the development of psychological distress. However, in the two more traditional and religious societies, the role of gender was less prominent. The gap between Ultra-Orthodox and Arab women, on the one hand, and secular women, on the other, might be explained in two possible ways. First, in traditional societies, women are very significant and influential in the communal and household spheres. Thus, in times of stress and crisis, the responsibility that they carry on their shoulders obligates them to act optimally and, as result, they do not allow themselves to be vulnerable. Moreover, as part of more traditional and collectivistic cultural contexts and regardless of their limited material resources, Ultra-Orthodox and Arab women are more directed toward self-transcendence, which means that their fundamental attitudes toward life involve less egotistic focus and more caring for others or for something greater than themselves (Wong, 2020a). This fundamental attitude protects them from psychological distress during stressful times. Second, as stated previously, the unique family structures of these two traditional societies, in which women are surrounded by many people, protects them from being lonely, depressed, and distressed. Religious and spiritual beliefs also strengthen feelings of being protected and empowerment among these groups of women. Additionally, leaders and authority figures in the Ultra-Orthodox community are in touch with the women of that community and provide encouragement, while secular women do not have such figures on whom to rely.

In the context of Arab society, this finding may be explained by the more traditional division of gender roles, in general, and in the home, in particular (Abu-Kaf, 2019; Haj Yahia, 2019; Abu-Kaf et al., 2020). During the lockdown, Arab men experienced difficulty staying at home with no clear chores or responsibilities. It seems that Arab men closed the gap in psychological distress levels with Arab women during the health pandemic and, especially, during the lockdown period.

### Study Limitations

Information about their experiences during the Covid-19 pandemic was provided only by the individual themselves and, therefore, the collected data are subjective. In addition, because we lack baseline information about the rates of psychological distress and resiliency factors among the surveyed individuals prior to the study period, we cannot with certainty ascribe the outcomes solely to the impact of the examined stressful situation.

Despite these limitations, the importance of this study lies in the fact that it is a field study carried during a stressful situation, which provided as a natural laboratory for the investigation of human behavior (Lazarus, 1982). It is also important to note that the secular Jewish, Ultra-Orthodox, and Arab communities are heterogeneous and include different subgroups. Future research should include large samples from the three populations and should pay more attention to variability within each population.

## Conclusion

The aim of this study was to evaluate stress and resiliency in three cultural groups in Israel. Through the lens of PP.2 the covid-19 pandemic enabled us to understand how a harsh situation of health pandemic, can lead to suffer, but then facilitate power, growth and strength. Our main results show that while the Ultra-Orthodox group exhibited resiliency, the two other groups (i.e., secular Jews and Arabs) suffered from major psychological distress. However, when we examined how the resiliency factors of SOC and hope explain the symptoms of psychological distress, similar results emerged among the three groups, with SOC having the strongest effect, followed by intrapersonal hope.

These results lead to important policy recommendations. Action must be taken to raise the awareness of decision-makers of the great importance of the mental well-being of residents during health crises. This aspect has been neglected in relation to other urgent issues, such as employment and economic and physical health. The allocation of resources including sense of coherence (Castiglioni and Gaj, 2020) for the improvement of existing mechanisms and strengthening the provision of mental-health services are critical tasks at this time.

## Data Availability Statement

The raw data supporting the conclusions of this article will be made available by the authors, without undue reservation.

## Ethics Statement

The studies involving human participants were reviewed and approved by the Human Subjects Ethics Committee of the Conflict Management and Resolution Program at Ben-Gurion University of the Negev (Approved Ethics Form No. 2020-008). The patients/participants provided their written informed consent to participate in this study.

## Author Contributions

OB-L wrote the manuscript, ran, and analyzed the data. SA-K and TK contributed to writing and analyzing the data. All authors contributed to the article and approved the submitted version.

## Conflict of Interest

The authors declare that the research was conducted in the absence of any commercial or financial relationships that could be construed as a potential conflict of interest. The handling editor declared a past co-authorship with one of the authors OB-L.
